# Postpartum Acquired Hemophilia Factor VIII Inhibitors and Response to Therapy

**DOI:** 10.5505/tjh.2012.68725

**Published:** 2012-06-15

**Authors:** Volkan Karakuş, Mustafa Çelik, Dilek Soysal, Bahriye Payzın

**Affiliations:** 1 Atatürk Research and Training Hospital, Departments of Internal Medicine 1st Division, Izmir, Turkey; 2 Atatürk Research and Training Hospital, Departments of Gastroenterology, Izmir, Turkey; 3 Atatürk Research and Training Hospital, Departments of Hematology, Izmir, Turkey

## TO THE EDITOR

Postpartum-acquired hemophilia A is a rare and potentially severe complication of pregnancy caused by an autoantibody against factor VIII [1]. Acquired factor VIII deficiency is associated with autoimmune conditions, neoplastic diseases, drug hypersensitivity, and pregnancy. A retrospective study analyzed 51 published cases of postpartum factor VIII inhibitors, with regard to the outcome according to treatment [2]. The annual incidence of acquired hemophilia is 0.2-1/1,000,000 and it is difficult to diagnose. Most patients present with major hemorrhage and the mortality rate is about 22% [3,4,5]. 

A 20-year-old female in the postpartum period presented to the hospital with vaginal hemorrhage that had begun 20 d earlier. Examination of the patient showed that there were no obstetrical or gynecologic pathologies, or acute or chronic diseases causing the hemorrhage. The patient’s laboratory data are summarized in the Table 1. The patient’s activated partial thromboplastin time (APTT) was 68.4 s. A mixing study using a 50:50 mixture of the patient’s plasma and control plasma showed no correction suggesting the presence of an inhibitor. The patient’s factor VIII inhibitor level was 2.5 BU mL^–1^ (normal: <0.6 BU mL^–1^). Fresh frozen plasma (174 units), factor VIII (1 unit of VIII:C kg–1 for every 2 percentage point increase in plasma VIII:C), and recombinant FVIIa (Novo-Seven 90 μg kg–1 IV bolus injection, and then the same dose every 3 h for 1 d) was administered; active bleeding was controlled after 2 d of the treatment. 

Intravenous immunoglobulin (IVIG, 400 mg kg–1 for 5 d) and IV methylprednisolone (60 mg d–1 for 1 week) was given (as recommended) to eliminate the inhibitor [6]. At follow-up 7 d after the initiation of treatment the patient was clinically stable with a normal aPTT. Methylprednisolone was initiated at 50 mg d^–1^ 8 d after the start of the initial treatment, and was then gradually tapered and stopped over the course of 6 weeks. The patient was discharged from the hospital 15 days after treatment was initiated. 

Factor VIII inhibitor was 0.55 BU 4 weeks after delivery. The patient’s postpartum hemorrhage was attributed to the presence of acquired factor VIII inhibitor. Factor VIII inhibitor can be seen in healthy pregnant women without a history of bleeding. Prolonged aPTT and normal PTT is the hallmark of laboratory diagnosis. The objectives of therapy are control of bleeding and elimination of the inhibitor [5,7]. Treatment strategies to control active bleeding include the use of factor VIII concentrates, activated prothrombin complex concentrates (anti-inhibitor coagulant complex, Feiba, Autoplex T), and recombinant human factor VIIa [6,8,9]. Elimination of factor VIII inhibitor requires the use of immunosuppressive modalities. A prospective randomized trial evaluated the efficacy of prednisone and cyclophosphamide alone, and the combination of both drugs in 31 non-hemophilic patients with factor VIII antibodies. All patients initially received prednisone (1 mg·kg^–1^·d^–1^ p.o.) for 3 weeks and the antibody disappeared in 10 of the 31 participants (32%) during the initial course of prednisone [10]. Another option for the treatment of acquired factor VIII inhibitors is administration of IVIG [11]. Written informed consent was obtained from the patient for publication. 

Acquired hemophilia A is a rare and often fatal disorder. Because of difficulties and misdiagnosis delays in diagnosis and treatment are common. As such, whenever acquired hemophilia A—with or without bleeding is suspected immediate consultation with a hemophilia experienced in the management of inhibitors should be initiated. 

**Conflict of Interest Statement **

The authors of this paper have no conflicts of interest, including specific financial interests, relationships, and/or affiliations relevant to the subject matter or materials included.

## Figures and Tables

**Table 1:**
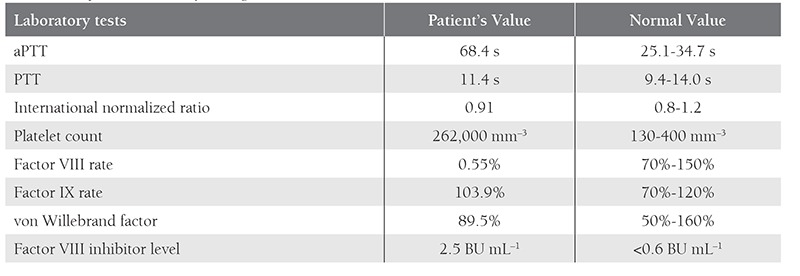
The patient’s laboratory findings.
